# Emotional abuse and depressive symptoms among the adolescents: the mediation effect of social anxiety and the moderation effect of physical activity

**DOI:** 10.3389/fpubh.2023.1138813

**Published:** 2023-06-27

**Authors:** Huiming Xu, Xuerong Luo, Yanmei Shen, Xingyue Jin

**Affiliations:** ^1^School of Physical Education, Changsha University of Science and Technology, Changsha, Hunan, China; ^2^Department of Psychiatry, National Clinical Research Center for Mental Disorders, and National Center for Mental Disorders, The Second Xiangya Hospital of Central South University, Changsha, Hunan, China

**Keywords:** childhood maltreatment, emotional abuse, depression, social anxiety, moderation, mediation

## Abstract

**Background:**

Childhood maltreatment has been identified as a risk factor for depressive symptoms. Social anxiety is closely associated with depression. Physical activity has been regarded as an underlying protective factor. Little is known about the complex relations among these factors in Chinese middle school students. This study aimed to explore whether social anxiety mediated the association between childhood maltreatment and depressive symptoms and investigate whether physical activity moderated the indirect or direct effect of the mediation model.

**Methods:**

A total of 1,570 middle school students were recruited and measured for childhood maltreatment (measured by Childhood Trauma Questionnaire—Short Form Chinese version), social anxiety (as the mediator, measured by the Chinese simplified version of Social Anxiety Scale for Adolescents), depressive symptoms (measured by the Chinese version of Depression Anxiety Stress Scales-21), physical activity (as the moderator), and covariates such as age, sex, and nationality. The proposed relationships were tested using mediation and moderated mediation models.

**Results:**

Emotional abuse was directly associated with depression, and the association between emotional abuse and depression was partially mediated by social anxiety. The associations between emotional abuse with depression and with social anxiety were moderated by physical activity.

**Conclusion:**

This study revealed the mediating role of social anxiety and the moderating role of physical activity between emotional abuse and depression, which emphasizes the potential benefits of sufficient physical activity to reduce social anxiety and depressive symptoms, and more intervention studies should be conducted to explore the direct influence of sufficient physical activity in the future.

## 1. Introduction

Childhood maltreatment (CM) is a kind of trauma that children and adolescents aged 0 to 17 suffered from their caregivers, often including physical, sexual, and emotional violence or neglect (1). CM is common worldwide but is often hidden; approximately three in four children, or nearly 300 million children aged 2–4 years worldwide, have regularly suffered one or more kinds of CM, such as physical punishment or emotional violence from their caregivers (2). Nearly 25% of adults reported suffering from physical abuse in their childhood (3, 4), 36% of them had suffered emotional abuse (5), 16% of them had suffered physical neglect (6), and 18% of them experienced emotional neglect (6).

Childhood maltreatment often leads to severe short-term and long-term consequences in the aspects of physical, sexual, and mental health (1). CM is closely associated with psychiatric disorders, with earlier onset age, severe symptoms, longer duration, more comorbidities, and worse response to treatment (7). Depression is a typical disease associated with CM. The investigation of Nelson et al. indicated that 45.59% of adults with depression reported at least one type of CM, and 19.13% of them reported more than one type of CM. People who suffered CM were 2.66 (for sexual abuse) to 3.73 (for emotional abuse) times more likely to get depression in adulthood, the mean age of the first depression onset was younger (23 *vs*. 27.1 years old), and more likely to be chronic (OR = 2.05) and treatment-resistant (OR = 1.90). The severity of depressive symptoms was most prominently associated with emotional abuse (8). A cohort study revealed that CM was significantly associated with depression relapse in the 2-year follow-up (OR = 1.035) (9).

Moderated mediation analysis is a useful method to determine relationships between variables. Mediators are in a causal sequence between two variables (10), and moderators influence the relationship between two variables conditionally (11). Previous studies have found some direct relationships between CM and depression; meanwhile, the underlying mediating (i.e., how CM is associated with depressive symptoms) and moderating mechanisms (i.e., when CM is associated with depressive symptoms) remain unclear. Therefore, to identify potential mediators and moderators of the process of CM to depression is necessary, which could offer insight into the prevention from maltreatment to potential depression and a possible way to reduce the negative impact of CM.

Social anxiety (SA) is prevalent in American adolescents and had a prevalence of 9.1% in 2010 (12). The peak age of onset for SA is during the teenage years (14.5 years old), and 50.9% of SA patients have onset as early as 14 years (13). SA and depression are highly comorbid (14), especially in adolescence (15), and SA is closely associated with major depression disorder (MDD) with an adjusted odds ratio of 3.9 for 12 months and an OR of 3.4 for a lifetime (16). Cummings et al. (15) indicated that SA symptoms often precede depressive symptoms. Moreover, proportional attributable fractions (PAFs) showed that 58.59% of depression and anxiety cases were potentially attributable to CM worldwide (17). CM significantly predicted SA (18). A meta-analysis also showed significant associations between CM and SA (19). The tripartite model of anxiety and depression hypothesized that anxiety and depression share a common component of negative affect (such as sadness, fear, and anger) and can contribute to their comorbidity (20). Individuals with SA and comorbid with MDD showed more childhood adversities (21). To sum up, we suppose that SA may serve as a mediator for CM and depression.

Physical activity (PA) is considered an important protective factor for depressive symptoms (22) in both men and women (23) and also in individuals with a high genetic vulnerability to depression (24). A systematic review suggested that compared with people without PA, people who exercise with the equivalent of 2.5 h of brisk walking each week were 25% less likely to get depression, and with the exercise of 1.25 h a week, the risk reduction was 18% (25). As for adolescents, PA and less sedentary behavior were significantly associated with a lower level of depression and greater life satisfaction and happiness in longitudinal and cross-sectional evidence (26). Felipe et al. (27) supported the claim that PA is an evidence-based treatment for depression. Previous research also showed significant associations between a higher level of PA and a lower level of SA symptoms (r = −0.12, p = 0.003) in cross-sectional studies, and PA interventions significantly lower SA symptoms (d = 0–0.22, *p* = 0.001) in longitudinal studies (28).

Taken together, CM, SA, and PA all play important roles in endorsing depressive symptoms. Still, the potential influence of these variables on depression is not clear among Chinese middle school students. Hence, this study aims to explore whether SA mediates the association between CM and depressive symptoms and evaluate the moderated role of PA. In this moderated mediation model, we hypothesized that SA might work as a mediator between CM and depressive symptoms, and PA plays a moderator role in the direct and/or indirect effects of CM on depressive symptoms.

## 2. Methods

### 2.1. Study population

In this cross-sectional study, by clustering sampling, students of age from 11 to 16 years in a middle school in Changsha, Hunan province, China, were invited to participate. The electronic questionnaires were created online by Wenjuanxing (www.wjx.cn), a professional Chinese online survey management tool, and distributed through the WeChat platform with the help of school teachers. Participants could scan the QR code or click the link of the questionnaire to fill in. All students and their parents have signed informed consent online. A total of 1,785 students were invited to participate; 55 students refused to participate and 160 students were excluded from the analysis due to incomplete data. A total of 1,570 students were included in the following analysis. The response rate was 87.96%. This study was approved by the ethics committee of the Second Xiangya Hospital of Central South University.

### 2.2. Measurement

#### 2.2.1. Childhood maltreatment

Childhood maltreatment was assessed by the self-reported scale Childhood Trauma Questionnaire—Short Form (CTQ-SF) Chinese version, translated from the English version of CTQ-SF, which was constructed by Bernstein et al. (29). CTQ-SF contains 28 items and assesses five kinds of CM including physical abuse, emotional abuse (EA), physical neglect (PN), emotional neglect (EN), and sexual abuse. Each item was measured on a 5-point Likert scale from “never happened (0)” to “always happened (4)” (30). Due to the difficulties of investigating sexual abuse in Chinese adolescents, only the former four kinds of CM (EA, PN, EN, physical abuse) were investigated and included in the following analysis. CTQ-SF was proven to have good reliability and validity in Chinese adolescents and college students (30, 31). In this study, Cronbach's alpha coefficient for the overall four subscales was 0.899 (alpha = 0.45 for PN, alpha = 0.81 for EA, alpha = 0.76 for physical abuse, and alpha = 0.91 for EN).

#### 2.2.2. Social anxiety

Social anxiety was assessed by the Chinese simplified version of the Social Anxiety Scale for Adolescents (SAS-A). Social Anxiety Scale for Adolescents (SAS-A) was compiled by La Greca and Lopez and contained 18 items (32). The Chinese simplified version of SAS-A contained 12 items and three dimensions of fear of negative evaluation, social avoidance and distress in new situations, and social avoidance and distress in general situations. Each item was measured on a 5-point Likert scale from “did not apply to me at all” (1) to “applied to me very much” (5). The higher the total score, the more severe the social anxiety. The Cronbach's alpha coefficient of the whole scale in this study was 0.92.

#### 2.2.3. Depressive symptoms

Depressive symptoms (called “depression” for simplicity) were assessed by the depression subscale of the Chinese version of Depression Anxiety Stress Scales-21 (DASS-21) (33). The original DASS was generated by Lovibond et al. (34). DASS-21 is a convenient scale utilized to measure the severity of depression, stress, and anxiety in recent 1 week, which contains 21 items. Each question was scored on a 4-point Likert from 0 (did not apply to me at all) to 3 (applied to me very much or most of the time) to measure severity and frequency (34). It has been widely used in adolescents (35), and the Chinese version of DASS-21 has good reliability and validity (33). The Cronbach's alpha coefficient of the depression subscale in this study was 0.90.

#### 2.2.4. Physical activities

Physical activities (PA) were measured by a question adapted from the Youth Risk Behavior Survey (YRBS) (36): “How many days did you get at least 60 minutes of moderate-intensity or vigorous-intensity (which increased your heart rate and made you breathe hard some of the time) physical activities in the past 7 days?” with options as 0, 1, 2, 3, 4, 5, 6, or 7 days a week. According to the WHO Global recommendations on physical activity for health, children and young people aged 5 to 17 should accumulate at least 60 min of moderate- to vigorous-intensity physical activity daily (37). Therefore, students who answered 7 days a week were considered as having sufficient PA, and others were considered as having low PA.

#### 2.2.5. Covariates

Demographic characteristics, such as age, sex, and nationality, were collected as covariates. Among these, 1,570 students (aged 13.10 ± 0.95) completed the whole questionnaire and were involved in the analysis; 833 (53.06%) were male students, 737 (46.94%) were female students, 1,490 (94.90%) were Han, and 80 (5.10%) were other nationalities.

### 2.3. Statistical analysis

**Figure 1:**
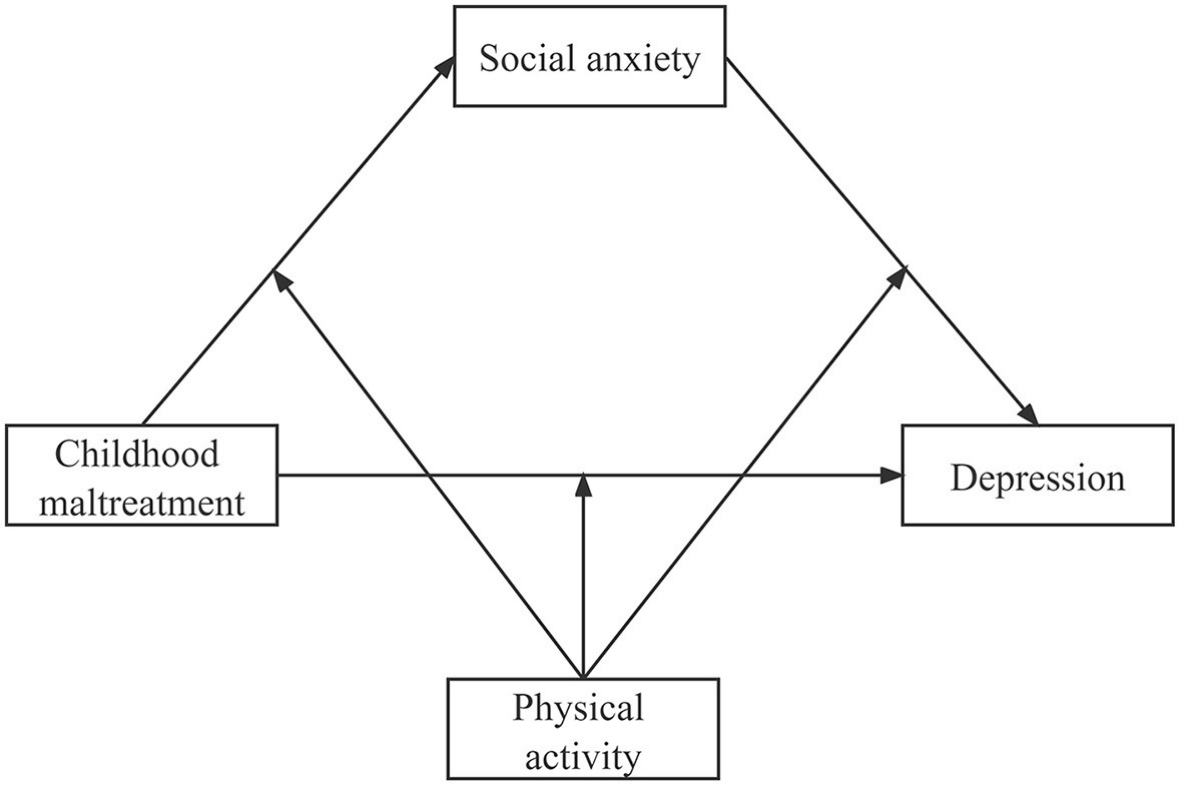
The proposed model.

All statistics were performed in SPSS (Version 25.0, Chicago, IL), and a value of *p* < 0.05 (two-tailed) was set as the significance level. Harman's single-factor test was conducted to assess common method bias. Spearman correlation analyses were used to analyze the correlations between variables such as CM, SA, depression, and PA. The moderated mediation analysis was conducted by the SPSS PROCESS 4.1 compiled by Hayes. The association between CM and depression mediated by SA was tested by model 4. The moderated effect of PA on the mediated model was tested by model 59 (Figure 1). Bootstrap analyses were conducted using 5,000 bootstrap samples and bias-corrected 95% confidence intervals. All continuous variables were normalized by Z-score normalization. Covariates, such as age, sex, and nationality, were controlled in all models.

## 3. Results

Among these 1,571 students, 326 (20.76%) students practiced sufficient PA for the past week, and 1,244 (79.24%) had low PA. The prevalence of depressive symptoms in this population of middle school students was 34.59% (543/1,570).

Harman's single-factor test showed that in the exploratory factor analysis, there were seven factors with characteristic roots greater than one. The first principal component explained 30.74% of the total variance, which was lower than the critical value of 40% (38). It indicated that there was no significant common method bias in this study.

**Figure 2:**
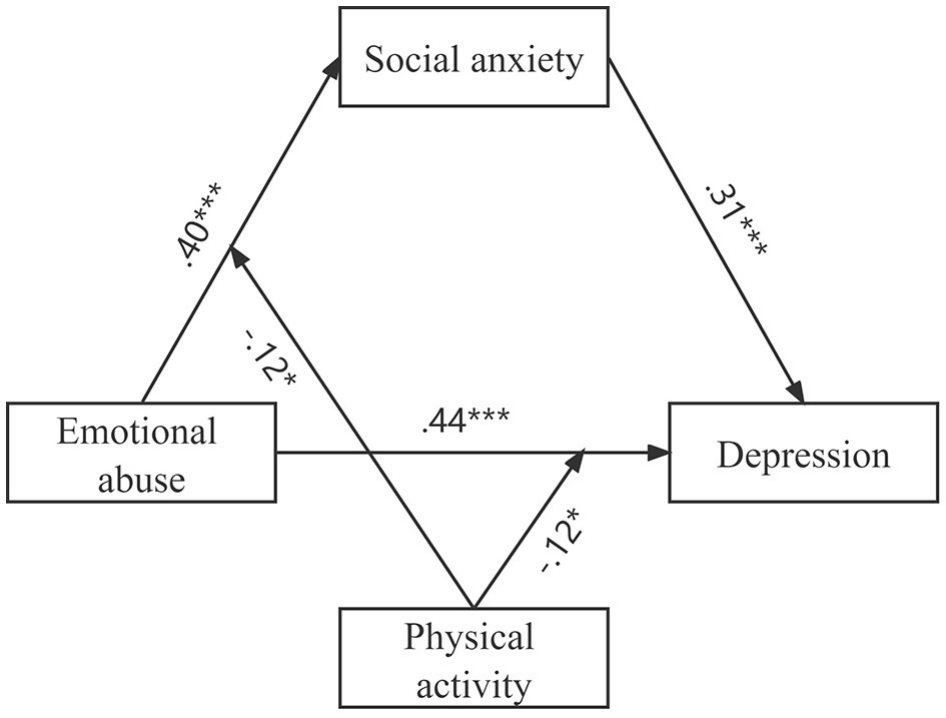
The final moderated mediation model.

After testing each subscale of CTQ-SF, we find one significant moderated mediation model as shown in Figure 2. The scores of scales measuring EA, SA, and depression are shown in Table 1. Correlation analysis shows that emotional abuse was significantly positively correlated with SA and depression and significantly positively correlated with PA. SA was significantly positively correlated with depression (Table 1).

**Table 1:** Descriptive statistics and correlations of the main study variables.

**Variables**	**M**	**SD**	**r**			
			**1**	**2**	**3**	**4**
1. Emotional abuse	7.49	3.56	1			
2. Social anxiety	32.61	14.10	0.42^***^	1		
3. Depression	4.16	4.68	0.55^***^	0.51^***^	1	
4. Physical activity	n	%	−0.05^*^	−0.04	−0.04	1
Low	1,244	79.24				
Sufficient	326	20.76				

M, mean; SD, standard deviation.

^*^p < 0.05, ^***^p < 0.001.

### 3.1. Mediation analysis

After controlling for covariates, such as age, sex, and nationality, the mediation effect test of SA shows a significant direct effect of EA on depression; SA played a significant role in mediating the relationship between them. Specifically, the total effect of EA on depression through SA was estimated as 0.56 (95% CI 0.52, 0.60), and the direct effect was estimated as 0.44 (95% CI 0.39, 0.48). EA was positively associated with SA (coeff = 0.40, 95% CI 0.36, 0.45, *p* < 0.001), and SA was also significantly positively associated with depression (coeff=0.31, 95% CI 0.27, 0.35, *p* < 0.001). The direct effect of EA on depression was statistically significant (coeff=0.44, 95% CI 0.39, 0.48, *p* < 0.001) (Table 2). After calculating with an online tool pwrSEM v0.1.2 (https://yilinandrewang.shinyapps.io/pwrSEM/), the mediation model showed a strong statistical power (power = 1.00) (39).

**Table 2:** Mediation effect of emotional abuse on depression (by parallel mediation analysis).

**Outcome variables**	**Social anxiety**			**Depression**		
	**Coeff**	**LLCI**	**ULCL**	**Coeff**	**LLCI**	**ULCI**
Constant	−0.33^*^	−0.58	−0.07	0.08	−0.14	0.30
Emotional abuse	0.40^***^	0.36	0.45	0.44^***^	0.39	0.48
Social anxiety				0.31^***^	0.27	0.35
Age	0.07^**^	0.03	0.12	−0.01	−0.05	0.03
Gender	0.24^***^	0.15	0.33	−0.08^*^	−0.16	−0.00
National	−0.03	−0.23	0.17	0.04	−0.14	0.21
	R-sq = 0.19, F_(4, 1, 565)_ = 89.51, *p* < 0.001			R-sq = 0.39, F_(5, 1, 564)_ = 200.68, *p* < 0.001		

LLCI, lower level of confidence interval; ULCI, upper level of confidence interval.

^*^p < 0.05, ^**^p < 0.01, ^***^p < 0.001.

### 3.2. Moderated mediation analysis

After controlling for covariates, the results of the moderated effect of PA are shown in Table 3. PA significantly moderated the direct effects of EA on depression (coeff = −0.12, 95% CI −0.22, −0.02) and the path EA to SA (coeff = −0.12, 95% CI −0.22, −0.01). But the moderating effect of PA in the path SA to depression was not significant (coeff = −0.01, 95% CI −0.11, 0.09).

**Table 3:** Moderated mediation analysis results for the relationship between emotional abuse and depression.

**Outcome variables**	**Social anxiety**			**Depression**		
	**Coeff**	**LLCI**	**ULCI**	**Coeff**	**LLCI**	**ULCI**
Constant	−0.26	−0.55	0.04	0.16	−0.10	0.41
EA	0.55^***^	0.41	0.68	0.59^***^	0.46	0.72
SA				0.32^***^	0.19	0.45
PA	−0.05	−0.16	0.06	−0.05	−0.15	0.04
EA^*^PA	−0.12^*^	−0.22	−0.01	−0.12^*^	−0.22	−0.02
SA^*^PA				−0.01	−0.11	0.09
Age	0.07^**^	0.03	0.12	−0.01	−0.05	0.03
Gender	0.24^***^	0.14	0.33	−0.09^*^	−0.17	−0.01
National	−0.03	−0.23	0.17	0.03	−0.14	0.21
	R-sq = 0.19, F_(6, 1563)_ = 60.69, *p* < 0.001			R-sq = 0.39, F_(8, 1561)_ = 126.91, *p* < 0.001		

EA, emotional abuse; SA, social anxiety; PA, physical activity; LLCI, lower level of confidence interval; ULCI, upper level of confidence interval.

^*^p < 0.05, ^**^p < 0.01, ^***^p < 0.001. The model was adjusted for age, sex, and nationality. All variables in the model were normalized.

**Figure 3:**
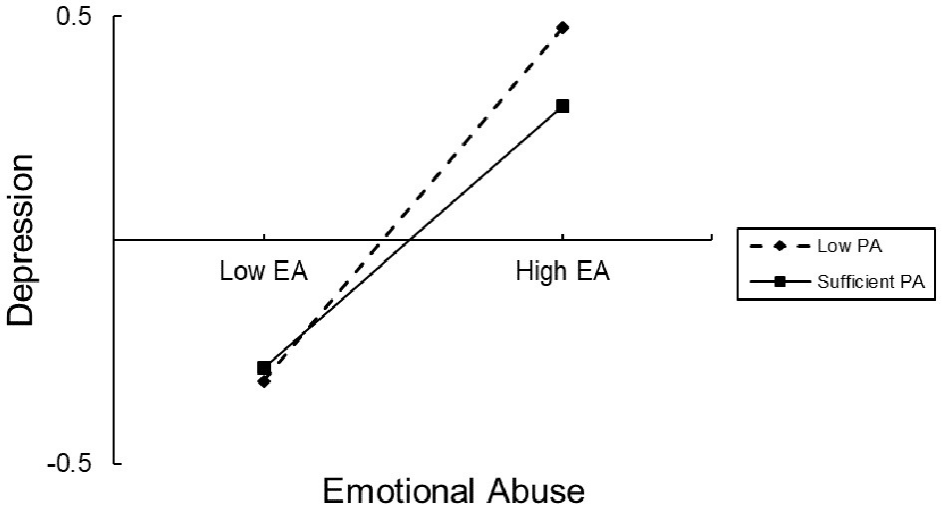
The conditional effect of emotional abuse (EA) on depression at the values of sports time. EA, emotional abuse; PA, physical activity.

**Figure 4:**
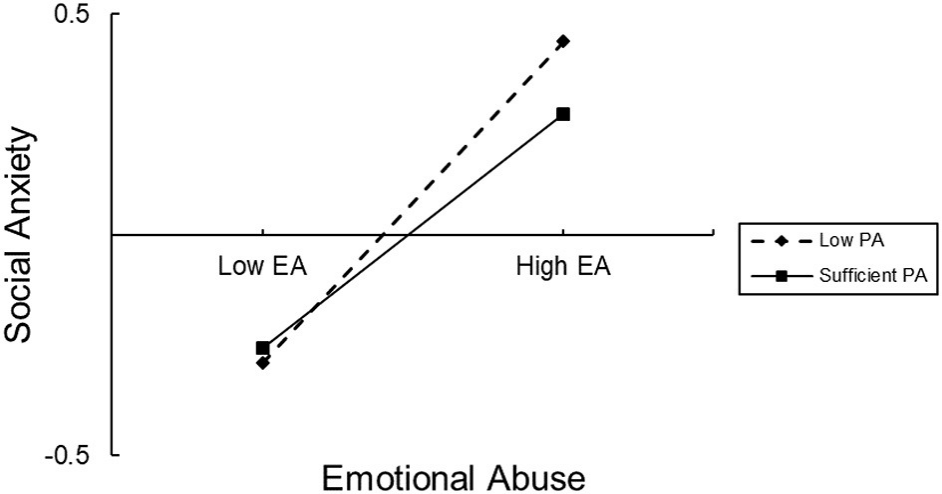
The conditional effect of emotional abuse (EA) on social anxiety at the values of sports time. EA, emotional abuse, PA, physical activity.

To test how the different levels of PA affected EA on depression, we divided PA into two levels, “low PA” and “sufficient PA”, according to the WHO standard (37). The direct impact of EA on depression was significant in both low PA (effect= 0.47, 95% CI 0.42, 0.52) and sufficient PA (effect=0.35, 95% CI 0.26, 0.43). For the indirect path, the impact of EA on SA was also significant in both low PA (effect = 0.13, 95% CI 0.10, 0.16) and sufficient PA (effect = 0.09, 95% CI 0.05, 0.14) (Table 4). Simple slope tests showed that the association between EA and depression was stronger for low PA than for sufficient PA (Figure 3). The association between EA and SA was also stronger for low PA than for sufficient PA (Figure 4). In other words, compared with low PA, sufficient PA may weaken the associations between EA and depression and between EA and SA.

**Table 4:** Conditional direct and indirect effects of emotional abuse on depression at different values of physical activity.

	**EA**→**DEP**			**EA**→**SA**→**DEP**		
	**Effect**	**BLLCI**	**BULCI**	**Effect**	**BLLCI**	**BULCI**
Low PA	0.46^***^	0.42	0.52	0.13	0.10	0.16
Sufficient PA	0.35^***^	0.26	0.43	0.09	0.05	0.14

EA, emotional abuse; SA, social anxiety; PA, physical activity; LLCI, lower level of confidence interval; ULCI, upper level of confidence interval.

^***^p < 0.001. The model was adjusted for age, sex, and nationality. All variables in the model were normalized.

## 4. Discussion

This study examined the mediating role of SA between CM and depression and explored the relationships among CM, SA, PA, and depressive symptoms via a moderated mediation model. These findings indicated that CM, SA, and PA were all significantly correlated to depressive symptoms, but the effects were different. SA mediated the association between EA and depressive symptoms. Moreover, the direct effect of CM on depressive symptoms and the path from EA to depressive symptoms was moderated by PA. The results support the importance of PA as a potential protective factor for adolescents to overcome the effect of EA on getting depressive symptoms and SA.

Different forms of EA, such as blaming, threatening, frightening, discriminating against, humiliating, or ridiculing (1), were related to internalizing behaviors in Chinese children (40), such as depression, anxiety, and suicide (41). LeMoult et al. also found that EA was strongly associated with MDD (OR 2.97, 95% CI 1.51, 5.82) (42). Many people who suffer from EA often have hopelessness, poor self-esteem, reduced sense of social support, and poor satisfaction with life (43). Previous studies found some mediators of CM to depression, such as attachment and anxiety (44), metacognitive beliefs impairment (45), perceived social isolation (46), and maladaptive strategies (47). A systematic review summed up as early maladaptive schemas, hopelessness, negative cognitive styles, brooding rumination, and overall emotion dysregulation were consistent psychological mediators of EA to depression (48). The experiences of EA were antecedents to forming a negative cognitive style (49–51), and negative cognition was an important feature for depression and SA. A classic cognitive-behavioral model of SA by Rapee and Heimberg indicated that people with SA often assume that other people are critical and may evaluate them negatively. SA attaches great importance to positive comment from others (52). When exposed to feared social situations and having negative self-beliefs, there was an imbalance between what the audience actually saw and what SA thought the audience might see. Once seeing external or feeling predicted negative evaluation indicators, it developed or maintained SA symptoms, showing the important effect of passive cognition on developing SA (52). This study showed the mediation effect of SA on EA to depressive symptoms; more studies are needed to explore further mechanisms between these relationships.

Consistent with previous studies, our study also showed the benefits of PA; to be specific, PA moderated the path from EA to depressive symptoms, and EA to SA, and the moderation effect of sufficient PA was stronger than low PA level. A meta-analysis of prospective cohort studies revealed that people with high levels of PA were less likely to develop depression (OR = 0.83) in youths, adults, and older adults (53). PA was considered to be an adjuvant therapy for SA and depression; after PA treatment, the SA symptoms and depressive symptoms were significantly lower (28, 54, 55). Traditional Chinese exercises, such as Tai Chi (56) and Baduanjin (57), have also been found to be effective in alleviating depression symptoms. PA could also improve self-esteem (58), improve social support (59), and reduce inferiority complex (60), which could help to overcome the negative cognitions caused by EA. Moreover, objective movement records could measure more information about PA than self-reported, such as PA patterns. In future studies, we should use the combination of self-reported activity and objective measures to a better record about PA. There are some more things we should consider, although we have found a weaker association between EA and depression at a sufficient PA level, a meta-analysis revealed that an excessive amount of PA was not so helpful, with only minor additional benefits and greater uncertainty (25).

In this study, we investigated CM and explored its association between SA, depression, and PA in a young population of children and adolescents aged 13.10 ± 0.95, showing their relationships in the early stages of children's development. Several limitations associated with this study should be noted. First, we cannot draw any causality between those factors in this cross-sectional study. Second, CM was collected retrospectively, which may introduce recall bias; sexual abuse, a significant form of CM, was not examined in this study; and future studies should endeavor to measure all of the five subtypes of CM. Third, the use of self-report scales to assess whether students meet the WHO PA standard missed more details about PA; future studies should utilize measurement tools that are more objective to improve the representativeness. Moreover, all data were collected from the same middle school in China, which makes it difficult to generalize to other samples. Additionally, though psychotherapy is a valuable intervention for depression, access to psychotherapy may be hard, and psychotherapy was not investigated in this study. Future research should also consider more potential confounding factors associated with depression, such as conditions of receiving psychotherapy, cognition styles, and bullying.

## 5. Conclusion

This study revealed the mediation effect of social anxiety on the development of emotional abuse to depressive symptoms in Chinese middle school students. Physical activity played a vital moderation role in the path from EA to SA and depression. For students who suffer from EA, it might be essential to get more sufficient PA to reduce SA and depressive symptoms. Getting enough PA is a possible efficient way to minimize the effects of emotional abuse, for both the general population and clinical populations as an additional non-drug treatment.

## Data availability statement

The raw data supporting the conclusions of this article will be made available by the authors, without undue reservation.

## Ethics statement

The studies involving human participants were reviewed and approved by the Ethics Committee of the Second Xiangya Hospital of Central South University. Written informed consent to participate in this study was provided by the participants and the participants' legal guardian/next of kin.

## Author contributions

XL, YS, and XJ were responsible for the study design. HX, YS, and XL were responsible for recruiting the participants. HX and XJ were involved in statistical analysis. HX wrote the first draft of the manuscript. XJ, XL, and YS wrote sections of the manuscript. All authors have contributed to the manuscript revision, read and approved the final manuscript, and agreed to submit it.
